# The plant Apolipoprotein D ortholog protects *Arabidopsis *against oxidative stress

**DOI:** 10.1186/1471-2229-8-86

**Published:** 2008-07-31

**Authors:** Jean-Benoit F Charron, Francois Ouellet, Mario Houde, Fathey Sarhan

**Affiliations:** 1 Département des Sciences biologiques, Université du Québec à Montréal, Case Postale 8888, Succursale Centre-ville, Montreal, Quebec, H3C 3P8, Canada; 2 Department of Molecular, Cellular & Developmental Biology, Yale University, 352 OML,165 Prospect St., New Haven, Connecticut, 06520-8104, USA

## Abstract

**Background:**

Lipocalins are a large and diverse family of small, mostly extracellular proteins implicated in many important functions. This family has been studied in bacteria, invertebrate and vertebrate animals but little is known about these proteins in plants. We recently reported the identification and molecular characterization of the first true lipocalins from plants, including the Apolipoprotein D ortholog *AtTIL *identified in the plant model *Arabidopsis thaliana*. This study aimed to determine its physiological role *in planta*.

**Results:**

Our results demonstrate that the AtTIL lipocalin is involved in modulating tolerance to oxidative stress. AtTIL knock-out plants are very sensitive to sudden drops in temperature and paraquat treatment, and dark-grown plants die shortly after transfer to light. These plants accumulate a high level of hydrogen peroxide and other ROS, which causes an oxidative stress that is associated with a reduction in hypocotyl growth and sensitivity to light. Complementation of the knock-out plants with the *AtTIL *cDNA restores the normal phenotype. On the other hand, overexpression enhances tolerance to stress caused by freezing, paraquat and light. Moreover, this overexpression delays flowering and maintains leaf greenness. Microarray analyses identified several differentially-regulated genes encoding components of oxidative stress and energy balance.

**Conclusion:**

This study provides the first functional evidence that a plant lipocalin is involved in modulating tolerance to oxidative stress. These findings are in agreement with recently published data showing that overexpression of ApoD enhances tolerance to oxidative stress and increases life span in mice and Drosophila. Together, the three papers strongly support a similar function of lipocalins in these evolutionary-distant species.

## Background

Lipocalins are small ligand-binding proteins found in bacteria and in invertebrate and vertebrate animals. Over 40 lipocalin members have been identified from all kingdoms [1]. They show a simple tertiary structure which gives them the ability to bind small, generally hydrophobic, molecules. Animal lipocalins play important roles in the regulation of immunological and developmental processes, and they are involved in the responses of organisms to various stress factors and in signal transduction pathways. It was recently shown that the insect glial *Lazarillo *lipocalin (GLaz) possesses a protective role against oxidative stress conditions and that its absence increases lipid peroxydation, reduces life span and accelerates neurodegeneration in *Drosophila *[2]. On the other hand, its overexpression protects against the effects of starvation, hypoxia and hyperoxia, and extends the fly's life span [3].

Data mining of genomic databases and bioinformatic predictions allowed us to determine that plants also possess lipocalins, which were classified as temperature-induced lipocalins (TILs) and chloroplastic lipocalins (CHLs) [4]. The *TIL *genes are induced by high and low temperature (LT) and their level of expression is associated with the plant's capacity to develop freezing tolerance (FT). Furthermore, the accumulation of TIL transcripts is limited to photosynthetic tissues and oscillates during the diurnal cycle. TILs do not show any targeting peptide, but the proteins are found at the plasma membrane. Sequence, structure and phylogenetic analyses revealed that TIL proteins share homology with three evolutionary related lipocalins: the bacterial lipocalin (Blc), the mammalian apolipoprotein D (ApoD), and the GLaz proteins.

In spite of the accumulating knowledge regarding the molecular features of plant lipocalins, very little is known about their function at the cellular and biochemical levels. However, the TIL properties, their association with the plasma membrane in photosynthetic tissues, and their accumulation in response to temperature stress support the hypothesis that these proteins may act as scavengers of potentially harmful molecules known to be induced by temperature stress and excess light [5]. In this report, we characterized knock-out, complementation and overexpression lines to determine the cellular and biochemical functions of the AtTIL lipocalin.

## Results and discussion

### AtTIL delays flowering and maintains leaf greenness

**Figure 1:**
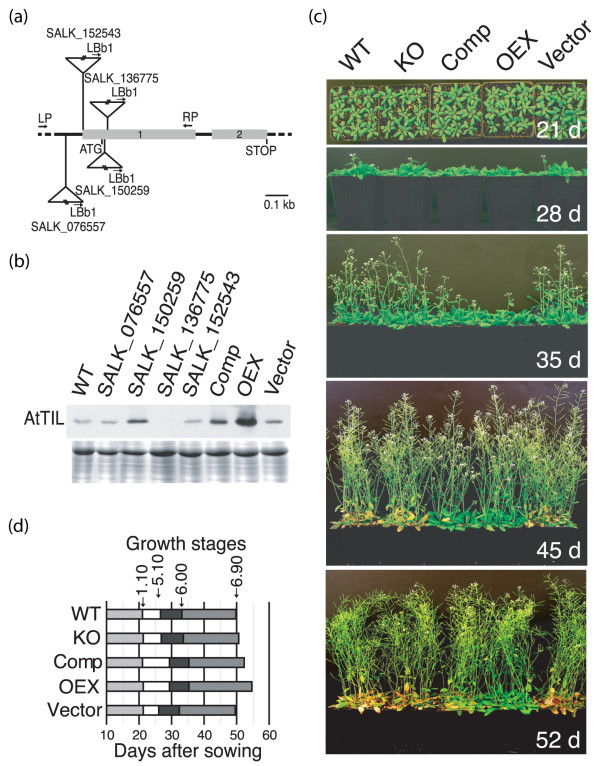
Modulation of AtTIL protein level affects development. **(a) **Genomic organization of *Arabidopsis *SALK lines carrying T-DNA insertions in the *AtTIL *gene [GenBank:BAB10998; Locus:At5g58070]. Boxes 1 and 2 are exons. The primers used for PCR analysis of the genomic DNA are indicated (see also Methods). **(b) **AtTIL protein levels in leaf extracts. Top panel: A rabbit anti-AtTIL antibody was used for immunoblot analysis. Bottom panel: Coomassie Brilliant Blue (CBB)-stained gel. **(c) **Plants were grown under normal conditions of temperature and photoperiod. Overhead (21 d) or lateral (28, 35, 45 d, 52 d) views are shown. **(d) **Developmental growth stages expressed according to Boyes *et al*. [31]. 1.10: 10 rosette leaves; 5.10: appearance of the first flower buds; 6.00: opening of the first flower; 6.90: completion of flowering. At least 30 plants per line per assay were monitored, and the experiment was repeated 3 times. WT, wild type Col-0 plants; SALK_XXXXXX, *AtTIL *T-DNA insertion lines from the SALK collection; Comp, SALK_136775 KO plant complemented by overexpression of *AtTIL*; OEX, an *AtTIL *overexpressing line; Vector, Col-0 transformed with a binary vector that does not carry the *AtTIL *cDNA (negative control).

**Figure 2:**
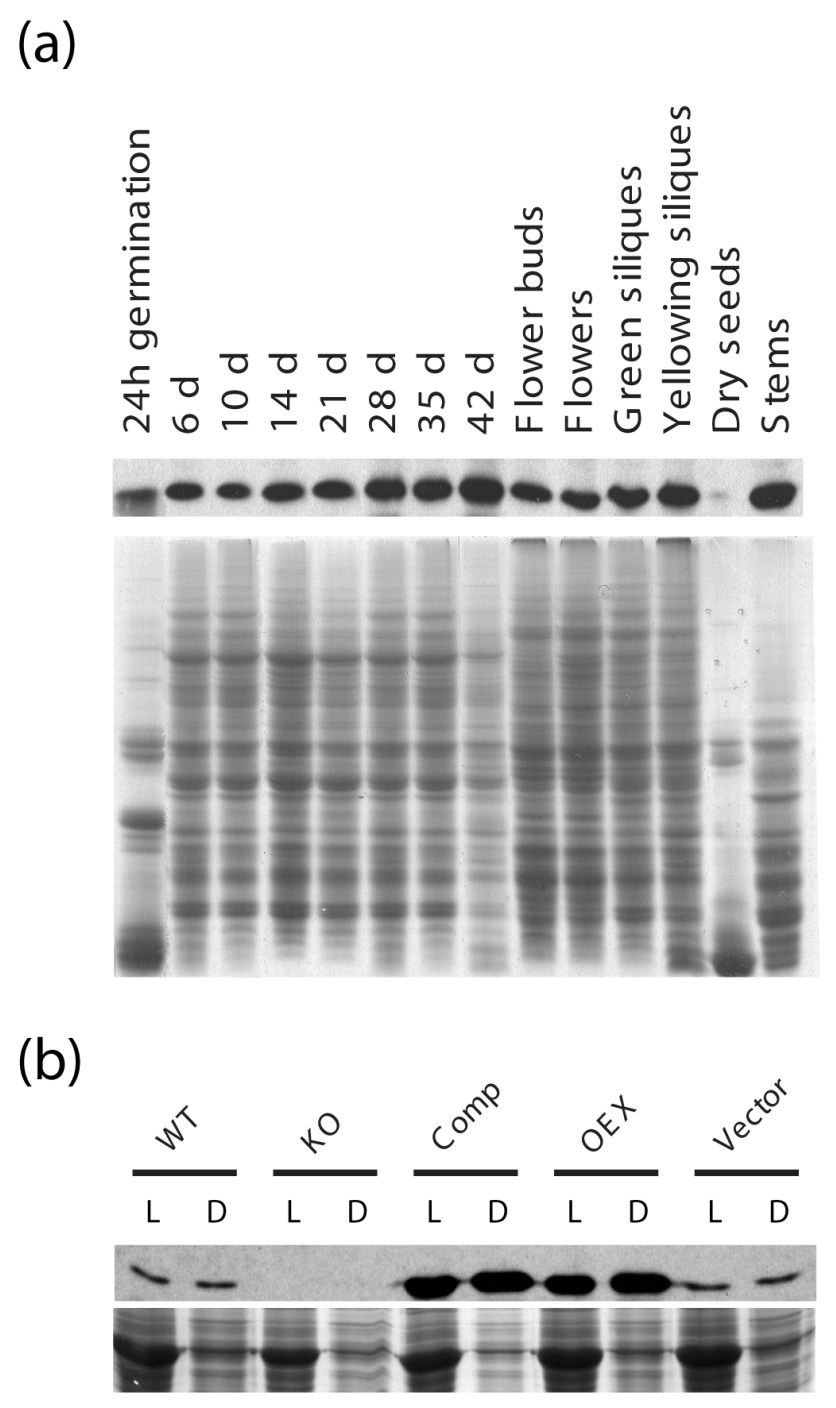
Accumulation of the AtTIL protein immunodetected with the anti-AtTIL antibody. **(a) **Proteins were purified from wild type plants grown under normal conditions, at various stages of their life cycle. **(b) **AtTIL protein levels in 7 day-old seedlings grown under light (L) or dark (D) conditions. Bottom panels: CBB-stained gel. WT, wild type Col-0 plants; KO, *AtTIL *T-DNA insertion lines from the SALK collection; Comp, KO plant complemented by overexpression of *AtTIL*; OEX, an *AtTIL *overexpressing line; Vector, Col-0 transformed with a binary vector that does not carry the *AtTIL *cDNA (negative control).

For functional analyses, four T-DNA lines carrying insertions in the *AtTIL *gene were analyzed to identify knock-out (KO) lines (Fig. 1a) [see Additional file 1] [6]. The SALK_136775 line carries a single insertion in the first exon of the *AtTIL *gene and shows no detectable AtTIL expression (Fig. 1b). Complementation of this KO line restored AtTIL protein accumulation to a level about two-fold higher than the wild-type (WT) plants (Fig. 1b, Comp). Overexpression of the *AtTIL *cDNA resulted in a 4-fold accumulation of the protein compared to the WT (Fig. 1b, OEX). Under normal growth conditions, the downregulation of *AtTIL *expression (KO) has no visible effect on plant growth and development compared to the WT (Fig. 1c). In contrast, the Comp and OEX lines show a delay in flowering and a stay-green phenotype clearly visible at 45 and 52 days, respectively (Fig. 1c and 1d, Table 1). These data suggest that an increased AtTIL level extends the vegetative phase and maintains leaf greenness. To determine if AtTIL protein accumulation is modulated during development, a western blot analysis was performed on samples prepared from tissues collected throughout the WT plant's life cycle. The results show that apart from the dry seeds stage, AtTIL is expressed at approximately the same level at all stages of development (Fig. 2a). Since there is no association between the protein accumulation and normal flowering, and because there is already a high level of protein before and during bolt elongation, we hypothesize that an excess of AtTIL may disrupt certain molecular events that ultimately delay the transition from the vegetative to reproductive phase.

**Table 1:** Rosette leaf number at time of bolting in wild-type and *AtTIL *mutant plants

WT	KO	Comp	OEX	Vector
9.9 ± 0.7	9.8 ± 0.8	11.0 ± 0.7 *	12.2 ± 0.8 **	10.0 ± 0.9

Values shown are the mean number ± SD of rosette leaves at the time of flowering. At least 12 plants of each line were used, and the experiments were repeated at least twice. Statistical analysis was performed by one-way ANOVA. The asterisks (**) and (*) indicate differences that are significant at the P < 0.001 and P < 0.01 levels respectively. Acronyms are as described in Fig. 1.

### AtTIL enhances tolerance to freezing and oxidative stress

**Figure 3:**
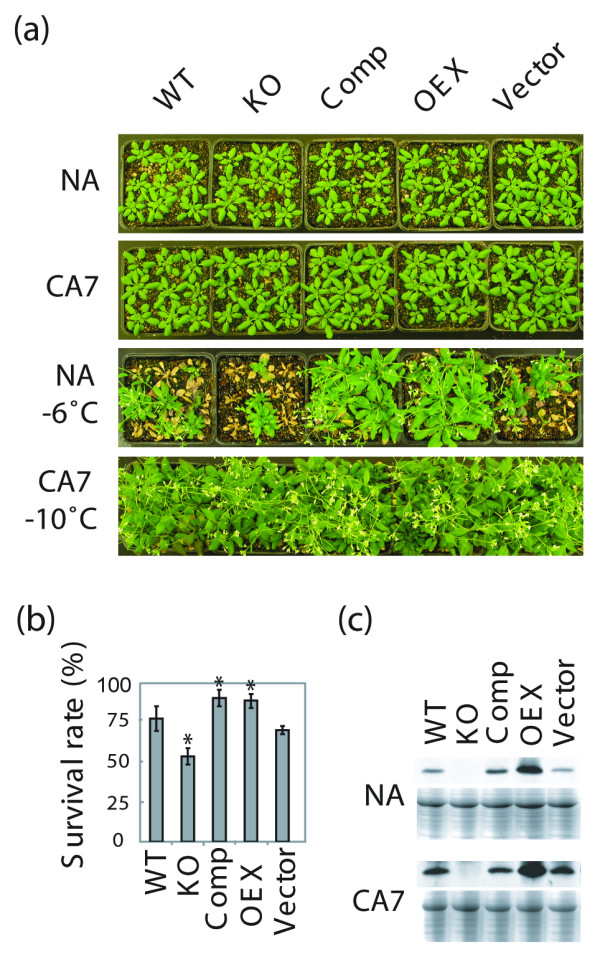
AtTIL enhances tolerance to freezing stress. **(a) **Plants were grown for 3 weeks at 22°C (NA) or grown at 22°C then transferred at 4°C for 7 days (CA7), and pictures were captured. The same plants were subjected to a freeze test performed at -6°C or -10°C for the NA and CA plants, respectively, and pictures were captured after a recovery period of 3 weeks. **(b) **Survival rate after freezing of NA plants to -6°C, expressed as a percent of surviving plants. Statistical analysis was performed by one-way ANOVA, and the asterisks (*) indicate differences that are significant at the P < 0.001 level. **(c) **AtTIL protein accumulation in leaves of NA and CA plants. Proteins were extracted and analyzed by immunoblotting. CBB-stained gels are shown as loading controls. Results are representative of at least three independent assays involving 18 plants per line per assay.

There are no noticeable morphological differences between the different *Arabidopsis *lines when they are compared before (non-acclimated; NA) or after cold acclimation (CA) (Fig. 3a). After subjecting NA plants to a freezing test at -6°C, a survival rate of 75% was obtained for the wild-type (WT) and control (vector) plants (Fig. 3b). In contrast, only 50% of the KO plants survived while the survival rate of the Comp and OEX plants was more than 90%. Freezing treatment caused typical damage and necrosis to the leaves of surviving WT and KO plants. On the other hand, leaves of OEX plants showed no damage after freezing and resumed vigourous growth when transferred to normal growth conditions at 20°C (Fig. 3a). After 7 days of cold acclimation (CA7), the level of AtTIL protein increases in all lines except in KO plants (Fig. 3c). This indicates that AtTIL accumulation is associated with an increased freezing tolerance that provides protection against damages caused by a sudden drop in temperature. On the other hand, the difference in sensitivity to freezing between NA WT and KO plants is not observed when plants are cold-acclimated prior to exposure to freezing (CA7 -10°C, Fig. 3a). Additional freezing tests performed down to -13°C did not reveal any differences between WT and KO plants [see Additional file 2], suggesting that cold acclimation is sufficient to provide maximal FT, and that AtTIL is likely not essential when plants undergo the full acclimation process. Protoplasts isolated from non acclimated lipocalin transgenic plants showed a higher survival rate compared to WT after freezing to -10°C. This increase in survival rate was associated with a decrease in the incidence of dehydration-induced injury [7].

**Figure 4:**
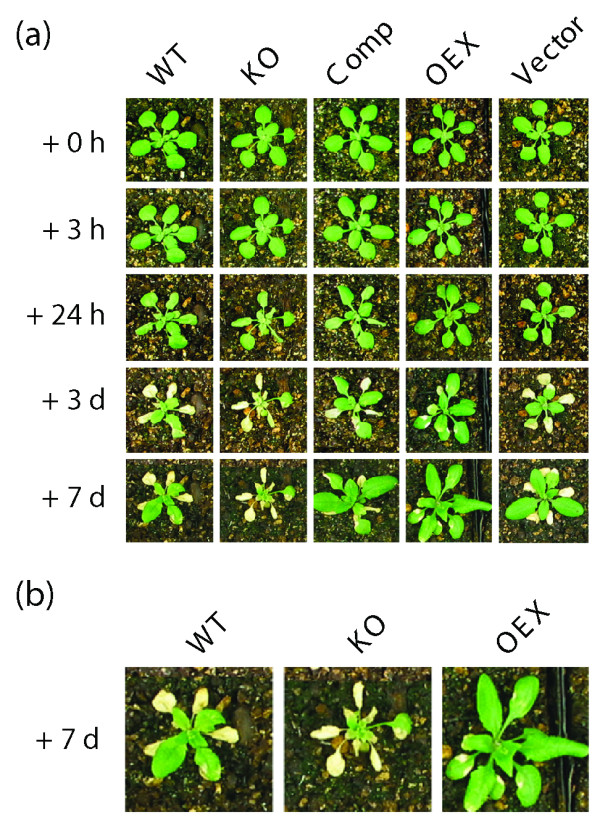
The level of AtTIL accumulation influences oxidative stress tolerance of *Arabidopsis*. **(a) **Plants were grown under normal conditions for 3 weeks and then sprayed until run off with either water or a 15 μM paraquat solution. Pictures were captured at the indicated time after treatment. Only the paraquat-treated plants are shown since no effect was observed for plants sprayed with water. **(b) **A close-up is shown to better show the necrotic lesions. Results are representative of at least three independent assays involving 32 plants per line per assay.

The freeze-induced lesions may be caused by harmful reactive oxygen species (ROS) generated when plants are exposed to freezing. To test the effect of ROS, plants were treated with the oxidant paraquat, a powerful herbicide that generates ROS [8-10]. After 7 days of treatment, KO plants showed more necrotic lesions than WT plants, whereas OEX plants were more resistant (Fig. 4a and 4b).

**Figure 5:**
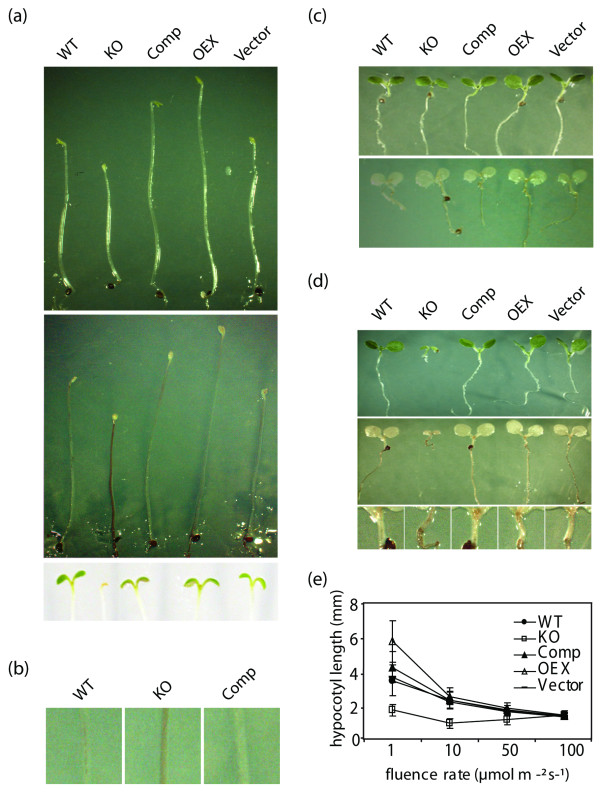
*AtTIL *knock-out plants show reduced hypocotyl elongation, hydrogen peroxide and other ROS accumulation. **(a) **Representative seedlings grown under dark conditions (upper panel), then stained with DAB (middle panel) to detect H_2_O_2 _and other ROS, or returned to light conditions for 24 h (lower panel). **(b) **Close-up of hypocotyls from dark-grown plants stained with DAB. **(c) **Representative seedlings grown under light-dark cycles (upper panel), then stained with DAB (lower panel). **(d) **Representative seedlings grown under continuous light (upper panel), then stained with DAB (middle panel). A close-up is shown to better show the DAB staining (lower panel). **(e) **Fluence response curves of seedlings grown under continuous light. Dark-adapted seedlings were exposed 7 days in the dark or under various intensities of continuous light. Images were captured and hypocotyl length was measured with the Image J software.

The effect of light stress on the different lines was monitored under different fluence rates (Fig. 5). Dark-grown KO plants show a reduction in hypocotyl elongation that is reversed in the Comp line, while OEX plants show longer hypocotyls (Fig. 5a, upper panel). There is no difference in AtTIL accumulation between plants grown under light or dark conditions (Fig. 2b). When dark-grown WT, Comp, OEX and control plants are transfered to normal photoperiod and light conditions, greening occurs and typical development resumes. In contrast, dark-grown KO plants transferred to light do not accumulate chlorophyll and die shortly after (Fig. 5a, lower panel). This suggests that the absence of lipocalin impairs the plants' ability to adapt to a sudden light exposure. There was no difference in growth and development when plants of the different lines were grown under a normal light-dark cycle (Fig. 5c, upper panel). Under a continuous moderate fluence of 100 μmol^-1 ^sec^-2^, the different lines show similar hypocotyl elongation but KO plants have smaller cotyledons (Fig. 5d, upper panel). These data indicate that in the absence of AtTIL, plants cannot tolerate the stress generated by continuous light and that a dark period is needed for plants to recover. Staining with 3,3'-diaminobenzidine (DAB) revealed that KO plants grown under stress conditions (dark or continuous light conditions) accumulate hydrogen peroxide and other ROS (Fig. 5a middle panel, Fig. 5b, Fig. 5d middle and lower panels) while KO plants grown under normal growth conditions do not (Fig. 5c lower panel). A differential growth sensitivity was observed when plants were grown under continuous low light. However, at moderate fluence of 50 and 100 μmol^-1 ^sec^-2^, the different lines show similar hypocotyl elongation (Fig. 5e), but the KO plants have small cotyledons as shown in Fig. 5D. It has been shown that hydrogen peroxide accumulation causes growth inhibition in maize and pine hypocotyls [11]. We speculate that the higher basal level of peroxide and other ROS in the KO plants is likely responsible for the higher sensitivity to oxidative stress, which leads to a reduction in hypocotyl growth and increased sensitivity to light. It was reported that the inability to eliminate excess peroxide increases sensitivity towards environmental stresses and induces the expression of several stress-related genes [12].

### Transcriptome analysis of *AtTIL *knock-out plants

Expression profiles were determined for WT and KO plants grown under NA and CA conditions (7 days at 4°C). No differences in gene expression were observed between the CA WT and CA KO, except for the absence of *AtTIL *transcript in the KO line. However, 66 genes were differentially regulated by more than 2-fold in the NA KO compared to the NA WT (51 up and 15 down-regulated; Table 2). Among these 66 genes, 49 are regulated in a similar manner (up or down) by LT exposure in WT plants while 5 are regulated in an opposite manner. These genes encode known or putative transcription factors or signal transduction proteins, heat shock proteins, enzymes involved in carbohydrate metabolism or oxidative stress responses, and senescence and circadian clock-associated proteins. The remaining 12 genes are regulated only in the KO line (* in Table 2; KO-specific genes) and encode proteins that are associated with stress responses. This analysis indicates that without exposure to LT, the absence of *AtTIL *mimicks part of the LT response. The accumulation of hydrogen peroxide and other ROS in the KO plants (Fig. 5) may act as a signal that triggers the changes observed in gene expression. A similar finding was reported for catalase-deficient plants under non-stressed conditions [13]. It is known that hydrogen peroxide accumulation results in differential gene expression [14].

**Table 2:** Genes showing at least two-fold differential expression (induction/repression) in *Arabidopsis AtTIL *knock-out plants.

			Differential expression (absolute values)					
			KO	KO 4°C	WT 4°C	KO 4°C		

			vs	vs	vs	vs	Regulation	

Putative Function and Reference	Locus ID	ProbesetIDs	WT	KO	WT	WT 4°C		

Upregulated Genes								

Transcription Factors:								
pseudo-response regulator 5 (APRR5)	At5g24470	249741_at	5,3	6,3	33,8	-1,0		†
zinc-binding family protein (DUF597)	At1g76590	259977_at	2,9	2,1	5,7	1,1	#	
WRKY family transcription factor (WRKY54)	At2g40750	257382_at	2,8	2,2	4,8	1,3	#	
DRE-binding protein (DREB1A)/CRT/DRE-binding factor 3 (CBF3)	At4g25480	254066_at	2,3	4,0	12,9	-1,4		
ABA-responsive element-binding protein (ABRE)	At1g49720	261613_at	2,2	2,4	4,9	1,1		
sigA-binding protein	At3g56710	246293_at	2,2	1,8	3,6	1,1	#	
heat shock factor protein, putative (HSF5) (HSTF5)	At1g67970	259992_at	2,1	2,2	4,0	1,2	#	
gigantea protein (GI)	At1g22770	264211_at	2,0	3,6	6,9	1,1		†
zinc finger (MYND type) family protein	At5g50450	248502_at	2,1	2,2	4,4	1,1		
zinc finger (C3HC4-type RING finger) family protein	At1g49230	260753_at	2,1	1,4	2,9	1,0	#	
CONSTANS-LIKE 13 zinc finger (B-box type) family protein	At2g47890	266514_at	2,1	3,5	6,5	1,1		
ABI3-interacting protein 1 (AIP1) (TOC1)	At5g61380	247525_at	2,1	3,1	5,4	1,2		†
Signal Transduction:								
invertase/pectin methylesterase inhibitor family protein	At5g62360	247478_at	5,2	1,3	5,8	1,1	#	
cold circadian rhythm-RNA binding 2; CCR2	At2g21660	263548_at	4,4	3,2	13,4	1,1		
acid phosphatase class B family protein	At2g39920	267361_at	4,0	-1,1	3,4	1,0	#	
auxin-regulated protein kinase, putative	At2g33830	267461_at	3,2	-1,6	1,7	1,1	*	
peptidyl-prolyl cis-trans isomerase/cyclophilin (CYP2)/rotamase	At2g21130	264019_at	2,3	2,9	6,4	1,1		
ankyrin repeat family protein (ACD6)	At4g14400	245265_at	2,2	2,0	4,5	-1,0		
protein kinase, putative BIK kinase	At3g55450	251789_at	2,2	2,1	4,2	1,1	#	
phosphorylase family protein	At4g24340	254163_s_at	2,1	-3,3	-1,3	-1,2	*	
Stress Reponse, Cell Rescue and Defense:								
DNAJ heat shock N-terminal domain-containing protein	At5g23240	249850_at	6,5	2,1	11,6	1,2	#	
DNAJ heat shock N-terminal domain-containing protein	At1g56300	256221_at	4,4	-1,2	3,1	1,2	#	
hydrophobic protein, low temperature and salt responsive protein	At4g30650	253627_at	2,3	7,8	17,5	1,0		
senescence-associated protein-related (SAG102)	At1g53885	262226_at	2,3	-4,7	-2,1	1,0		
hydrophobic protein, low temperature and salt responsive protein	At4g30660	253581_at	2,1	-2,7	-1,1	-1,2	*	
universal stress protein (USP) family protein	At3g62550	251221_at	2,0	-2,4	-1,3	1,1	*	
heavy-metal-associated domain-containing protein	At1g51090	245749_at	2,0	5,7	11,1	1,0		
pathogenesis-related thaumatin family protein (calcium storage)	At1g20030	261248_at	2,0	-1,7	1,0	1,0	*	
cold-regulated protein (cor15b)	At2g42530	263495_at	2,0	24,0	47,7	1,0		
pathogen and circadian controlled 1 (PCC1)	At3g22231	256766_at	2,0	1,6	2,9	1,0	#	
Metabolism:								
riboflavin biosynthesis protein, putative	At2g22450	264045_at	2,8	1,2	3,2	-1,0	#	
pyruvate decarboxylase, putative	At5g54960	248138_at	2,6	1,6	3,7	1,1	#	
preprotein and amino acid transporter	At4g26670	253981_at	2,4	1,8	4,0	1,1	#	
succinate-semialdehyde dehydrogenase (SSADH1)	At1g79440	262892_at	2,2	2,6	5,6	1,0		
aldose 1-epimerase family protein	At3g47800	252387_at	2,2	1,6	3,7	-1,1	#	
nucellin protein, putative	At4g33490	253331_at	2,1	-1,1	1,7	1,1	*	
auxin-responsive family protein	At5g35735	249719_at	2,1	1,2	2,4	1,0	#	
acyl CoA:diacylglycerol acyltransferase (DGAT)	At2g19450	267280_at	2,1	3,0	5,9	1,1		
isoamylase, putative/starch debranching enzyme, putative	At4g09020	255070_at	2,1	3,0	6,0	1,0		
2-oxoacid-dependent oxidase, putative (DIN11)	At3g49620	252265_at	2,1	-2,8	-1,2	-1,1	*	
OEP16-like protein	At2g28900	266225_at	2,1	2,7	5,8	-1,0		
starch phosphorylase, putative	At3g46970	252468_at	2,1	1,6	2,9	1,1	#	
glycoside hydrolase starch-binding domain-containing protein	At5g26570	246829_at	2,0	2,5	4,9	1,0		
amino acid permease family protein or GABA permease	At2g01170	265790_at	2,0	1,1	2,1	1,0	#	
Expressed Protein								
expressed protein	At4g16146	245319_at	4,0	4,2	14,7	1,2		
expressed protein	At1g53035	261318_at	2,6	5,1	12,0	1,1		
expressed protein	At4g04330	255331_at	2,6	-1,6	1,5	1,1	*	
expressed protein	At2g14560	265837_at	2,5	2,9	6,1	1,2		
expressed protein	At2g15890	265478_at	2,4	-3,0	-1,4	1,1	*	
expressed protein	At1g70420	264314_at	2,1	-1,8	1,6	-1,3	*	
expressed protein	At1g14870	262832_at	2,1	4,0	5,9	1,4		

Downregulated Genes								

Transcription Factors:								
late elongated hypocotyl (LHY)	At1g01060	261569_at	-3,2	6,2	2,1	-1,1		†
CONSTANS-LIKE 2 (COL2)	At3g02380	258497_at	-2,4	-2,0	-3,2	-1,5	#	†
Signal Transduction:								
cysteine proteinase, putative	At2g27420	265665_at	-2,3	2,3	1,0	-1,0	*	
calcium-binding RD20 protein (RD20)	At2g33380	255795_at	-2,1	-2,1	-3,7	-1,2	#	
Stress response, Cell Rescue and Defense:								
*At *TIL, lipocalin	At5g58070	247851_at	-3,6	-1,7	1,5	-8,8	*	
putative membrane related protein CP5	At1g55960	260603_at	-2,2	-2,0	-4,3	-1,0	#	
phosphate-responsive protein, putative (EXO)	At4g08950	255064_at	-2,0	-2,0	-5,3	1,3		
Metabolism:								
dehydrodolichyl diphosphate synthase, putative	At5g58770	247780_at	-3,1	-2,8	-7,6	-1,1		
trehalose-6-phosphate phosphatase (TPPA)	At5g51460	248404_at	-2,8	-2,6	-6,7	-1,1		
starch synthase, putative	At1g32900	261191_at	-2,5	6,4	2,7	-1,1		
carbonic anhydrase family protein	At3g52720	252011_at	-2,2	-3,3	-6,9	-1,1		
glutathione S-transferase, putative (ERD9)	At1g10370	264436_at	-2,2	4,5	2,1	-1,0		
adenosylmethionine decarboxylase family protein	At5g15950	246490_at	-2,0	6,6	3,5	-1,1		
Expressed Protein								
expressed protein	At3g28270	256603_at	-2,3	-1,9	-4,0	-1,1	#	
expressed protein	At1g68440	259856_at	-2,1	-2,5	-4,6	-1,1		

* 12 genes regulated (induced or repressed) only in the KO line (not regulated by exposure of the WT to LT)

# 22 genes for which the regulation in KO is at least 50% of the regulation seen in WT exposed at 4°C

† key regulators of the circadian clock

Several genes related to the disease resistance pathway are overexpressed in the *AtTIL *KO plant. The WRKY54 gene is known for its association with the defense response and involves an increase in salicylic acid (SA), which accumulates during oxidative stress and mediates the induction of defense response genes [15,16]. Similarly, the SigA binding protein is a homologue of MSK1 which was shown to cause an accumulation of SA [17]. The At3g55450 protein is most homologous to the BIK1 kinase which is induced by ROS generators such as paraquat and proposed to play a role in regulating optimal SA levels [18]. Induction of the BIK1 kinase-like gene in *AtTIL *KO plants without exposure to any stress suggests that the level of ROS is higher than normal in these plants. The greater susceptibility to paraquat is also indicative that the *AtTIL *KO plants are less capable of fighting additional oxidative stress. Furthermore, the up-regulation of riboflavin biosynthesis protein in KO plants suggests that riboflavin becomes limiting when *AtTIL *is absent. Since riboflavin deficiency is associated with oxidative stress and is an important component of FAD-requiring enzymes such as glutathione reductase, it is possible that KO plants may try to fight oxidative stress by increasing their level of riboflavin.

The microarray analysis suggests that in the KO plants, starch synthesis is reduced while starch catabolism is accelerated to provide additional soluble sugars needed to fight oxidative stress. In addition, the Krebs cycle is inhibited under stress conditions, preventing the efficient utilization of the glycolysis products and thus affecting the respiratory machinery [19]. To overcome this situation, plants rely on the γ-aminobutyrate (GABA) shunt. Succinic semialdehyde dehydrogenase, the last enzyme of the GABA shunt, is upregulated in *AtTIL *KO plants. The GABA shunt is a bypass of the Krebs cycle which provides succinate and NADH to the respiratory machinery [20]. In plants, the activity of this pathway is enhanced in response to biotic and abiotic stresses [21,22] suggesting a potential role in reducing the impact of oxidative stress in mitochondria [19].

Animal lipocalins have been suggested to have a role in restoring membrane integrity caused by oxidative stress, likely by their binding to fatty acids [23]. This study provides evidence that the plasma membrane-associated AtTIL lipocalin plays a role in protecting plants against the oxidative stress induced by freezing, paraquat treatment and light. We hypothesize that during oxidative stress, lipocalins may bind and scavenge peroxidated lipids, and thus help restore membrane integrity. Our findings are in agreement with recently published data showing that overexpression of ApoD enhances tolerance to oxidative stress and increases life span in mice and Drosophila [24,25]. The available evidence therefore point towards a universal cellular function of lipocalins among species.

## Methods

### *AtTIL *lines

*Arabidopsis *ecotype Columbia (Col-0) was the genetic background of all the lines used. T-DNA knock-out (KO) lines for the *AtTIL *gene were obtained from the Salk Institute Genome Analysis Laboratory . Seeds were sown on agar plates containing 50 μg/ml kanamycin, stratified for 2 days at 4°C and grown at 22°C. Kanamycin-resistant plants were propagated as individual lines on potting medium consisting of two parts *Arabidopsis *growing medium PM-15-13 (LEHLE seeds), one part vermiculite and one part black earth. Based on the segregation analysis of plants grown on antibiotic-containing plates, lines putatively homozygous for the T-DNA insertion were subjected to PCR analysis using T-DNA-specific (LBb1) and *AtTIL*-specific (RP) primers, and the resulting PCR fragments were sequenced to determine the precise insertion sites.

To generate the complementation (Comp) and overexpressing (OEX) lines, the *AtTIL *ORF was first cloned into pRTL2, a vector that contains a double cauliflower mosaic virus (CaMV) 35S promoter and a 35S terminator [26]. The resulting PRO_35S_:*AtTIL*:TERM_35S _cassette was then cloned into the binary plant expression vector pPZP121 [27] and electroporated into *Agrobacterium *GV3101. KO and WT *Arabidopsis *plants were transformed with this vector using the floral dipping method [28] to generate the Comp and OEX lines, respectively. Two independent OEX lines were selected for complete characterization. Since similar results were obtained, only one line is presented. To generate a negative control, WT plants were transformed with the pPZP121 vector that does not contain the *AtTIL *cassette. Transformed plants of all lines were selected by growth on gentamycin-containing medium. Homozygous plants of the fourth generation after transformation were used for the experiments.

### Plant growth conditions and treatments

Surface-sterilized seeds were sown on sterile half strength Murashige and Skoog medium (Sigma-Aldrich). Seeds were stratified for 2 days at 4°C then germinated and grown at 22°C/18°C (day/night) with a 16 hr photoperiod and a photon flux density of 90 μmol m^-2 ^sec^-1^. For soil-based analyses, seeds were sown directly on potting medium, stratified for 3 days at 4°C and grown for 11 days at 20°C under long day conditions (16 hr light/8 hr dark) at a photon flux density of 90 μmol m^-2 ^sec^-1^. Seedlings were then transferred to 3.5 inches or 1.5 inches pots containing potting medium and grown under the same conditions as described above.

For low temperature treament, 3 week-old soil-grown plants were transferred to 4°C for 7 days under the same photoperiod conditions. For paraquat treatment, paraquat (Sigma) was dissolved in water at a concentration of 15 μM and plants were sprayed once with this solution until run off. Control plants were sprayed with distilled water. After spraying, plants were grown for 7 days under normal conditions of temperature and photoperiod.

### Determination of freezing tolerance

A Caltec Scientific Ltd. Model 8–792 Large Capacity Temperature Stress Chamber was used to perform the freezing tolerance tests. This instrument consists of four major component systems: a Sanyo Model MDF-792 24.75 ft^3 ^capacity ultra-low temperature chest freezer, a custom designed stainless steel plenum box with its integral blower and heater (provides air circulation and heating) and an Omega Engineering Inc. Model CN3002 programmable profile controller (monitors the test-chamber air temperature and controls the heating element). The controlled action of the heater combines with the constant cooling of the freezer to achieve the desired temperature at any given time.

Non-acclimated (NA) and cold-acclimated (CA) soil-grown plants (3 weeks-old) were subjected to the following freezing regime. The plants were equilibrated at -1°C for 2 hr and were seeded with ice chips to initiate freezing. The temperature was then lowered gradually (2°C per hr) to -6°C and maintained at this temperature for 6 hr. The temperature was then gradually increased (2°C per hr) to 4°C. To determine temperature variability in the freezer, temperatures were monitored by 6 independent thermocoupled T probes distributed in the freezer and connected to an Agilent 3497-0A data acquisition/switch unit. Freezing regimes that showed more than 0.5°C discrepancies between the different probes were rejected. To minimize light stress effects after the freezing treatment, plants were thawed at 4°C for 24 hr in the dark and away from direct light in the growth chamber (20°C) for an additional 24 hr before returning to normal light conditions. Pictures were taken 3 weeks after the freezing treatment. Eighteen plants were frozen per line per assay, and the experiment was repeated 3 times with independent biological replicates.

### Hypocotyl analyses

Seeds were sown on sterile half strength Murashige and Skoog medium (Sigma-Aldrich) and stratified in the dark at 4°C for 6 days. They were then exposed to white light (90 μmol m^-2 ^s^-1^) at 20°C for 60 min and returned to darkness for 23 hr before being exposed to varying fluence rates at 20°C for 7 days. Hypocotyl length was measured using the Image J 1.36b software (mean ± SD; *n *= 40). H_2_O_2 _and other ROS were detected *in situ *by 3,3'-diaminobenzidine (DAB) staining as described [29].

### Protein isolation and immmunoblot analysis

The antibody raised against the wheat TaTIL lipocalin protein (4) does not cross react with the Arabidopsis AtTIL lipocalin, therefore it was necessary to raise a specific antibody against the latter. The *AtTIL *cDNA was cloned in the pTrc-HIS vector (Invitrogen) and electroporated into the E. coli strain DH5α. The recombinant HIS::AtTIL protein was produced by induction with 1 mM IPTG for 3 hr. The cells were collected, broken by lysozyme treatment and sonication, and HIS-tagged proteins were purified by immobilized metal affinity chromatography on Ni-NTA agarose (Novagen). The purified proteins were more than 90% pure and used to immunize a rabbit to obtain polyclonal antibodies.

Aerial parts of the Arabidopsis plants were cut and immediately frozen in liquid nitrogen. One hundred milligrams of leaf material was processed as one sample. Proteins were isolated using Tri Reagent according to the manufacturer's instructions (Sigma). Samples were separated on 12% SDS-PAGE gels and the rabbit anti-AtTIL antibody (1:10,000) was used for the immunoblot analysis. Detection was performed with a peroxidase-coupled anti-rabbit IgG secondary antibody (1:25,000) and the Western Lightning Chemiluminescence Reagent Plus (Perkin-Elmer).

### PCR and Southern blot analyses

Genomic DNA was extracted from flower buds of the different Arabidopsis lines. PCR analysis was performed according to the SIGnAL protocol using the recommended primers designed with the SIGnAL iSect Primer Design sofware: LBb1 (5' GCGTGGACCGCTTGCTGCAACT 3'), LP (5' CTGGATCCAGAGATGAAGTCG 3') and RP (5' AAGACGTGTATGGTACCGTCG 3'). For Southern analysis, the DNA (5 μg) was digested by PciI (New England Biolabs) and fractionated by electrophoresis on a 0.7% agarose gel. After electrophoresis, the gel was transferred to a positively charged nylon membrane and hybridized with 32P-labeled probes corresponding to the T-DNA of pROK2 (vector used to generate the SALK lines) or the T-DNA of the 35S:*AtTIL *vector. The following primer pairs were used for amplification of the fragments used as probes: 5'-CAGCAAAATCACCAGTAGCACCATTACCAT-3' and 5'-GCGATAGAAAACAAAATATAGCGCGCAAAC-3' for the pROK2 T-DNA, and 5'-ACGAAACGTGGAGCAACGGGAAGAG-3' and 5'-TGCACATACAAATGGACGAACGGATAAACC -3' for the *AtTIL *T-DNA. All filters were washed at high stringency (0.1× SSC, 0.1% SDS), exposed to K screens (Kodak) and analyzed on a Molecular Imager FX (Bio-Rad).

### Transcriptome analysis

Non-acclimated and cold-acclimated WT and KO plants were used for microarray analysis. For each sample, three independent biological replicates of 25 plants were harvested for RNA isolation. RNA quality assessment and hybridization to the Affymetrix GeneChip *Arabidopsis *ATH1 Genome Arrays were performed at the McGill University and Génome Québec Innovation Centre (Montreal QC Canada). Data accumulation and analysis were performed using the Robust Multi-array Average analysis (RMA) [30], and data was deposited in the ArrayExpress database [ArrayExpress:E-TABM-430]. An analysis of variance was performed using GraphPad InStat 3 to select genes that are significantly differentially expressed by at least two-fold under the conditions specified for each analysis (4°C vs 22°C and KO vs WT). Functional classification of the genes was done according to their annotation in the TAIR database , by BLAST search of the NCBI database  and by literature search performed using the gene name and annotation. RT-PCR analyses of *AtTIL *and randomly selected transcripts were performed to confirm the absence of *AtTIL *expression in the KO plants and to validate the microarray results [see Additional file 3].

## Abbreviations

ApoD: Apolipoprotein D; CA: cold acclimation; Comp: complementation; DAB 3: 3'-diaminobenzidine; FT: freezing tolerance; GLaz: insect Lazarillo protein; KO: knock-out; LT: low temperature; NA: non-acclimated; OEX: overexpression; ROS: reactive oxygen species; TIL: temperature-induced lipocalin; WT: wild-type.

## Authors' contributions

JB performed all experimental procedures; MH performed the microarray analysis; JB and FS conceived the study; JB, FO, MH and FS participated in the experimental design and wrote the manuscript. All authors have read and approved the final manuscript.

## Supplementary Material

Additional file 1 Genomic organization of *Arabidopsis *SALK lines carrying T-DNA insertions in the *AtTIL *gene (At5g58070). **(a) **PCR analysis of genomic DNA extracted from the different lines, performed with primers indicated in Fig. 1. **(b) **Southern blot analysis of genomic DNA extracted from the different lines. Left panel, probe detecting the pROK2 T-DNA insertion present in the SALK lines; right panel, probe detecting both a portion of the *AtTIL *transgene T-DNA (including the right border) and the *AtTIL *endogenous gene. WT, wild type Col-0 plants; SALK_XXXXXX, *AtTIL *T-DNA insertion lines from the SALK collection; Comp, SALK_136775 KO plant complemented by overexpression of *AtTIL*; OEX, an *AtTIL *overexpressing line; Vector, Col-0 transformed with a binary vector that does not carry the *AtTIL *cDNA (negative control). Click here for file

Additional file 2 AtTIL does not enhance tolerance to freezing stress in cold acclimated plants. Plants were grown for 3 weeks at 22°C then transferred at 4°C for 7 days (CA7). The plants were subjected to a freeze test performed at various temperatures and pictures were captured after a recovery period of 3 weeks. Results are representative of at least two independent assays involving 11 plants per line per assay. Click here for file

Additional file 3 Validation of microarray data using reverse transcriptase-PCR (RT-PCR). Transcript levels of randomly selected genes were quantitated in the control and cold-treated WT and *AtTIL *KO plants by RT-PCR. *UBQ10 *was used as a constitutively expressed control transcript. Reactions were performed using three independent biological replicates. Only one replicate is presented. At1g32900, starch synthase; At3g52720, carbonic anhydrase; At5g51460, trehalose-6-phosphate phosphatase; At2g01170, amino acid permease; At1g79440, succinate-semialdehyde dehydrogenase; At2g19450, diacylglycerol O-acyltransferase; At4g09020, isoamylase; At5g54960, pyruvate decarboxylase. Click here for file
