# Supplementary figures and images for: The plant Apolipoprotein D ortholog protects Arabidopsis against oxidative stress
Source: BMC Plant Biol. 2008 Jul 31;8:86. doi: 10.1186/1471-2229-8-86 (PMC2527315; doi:10.1186/1471-2229-8-86)

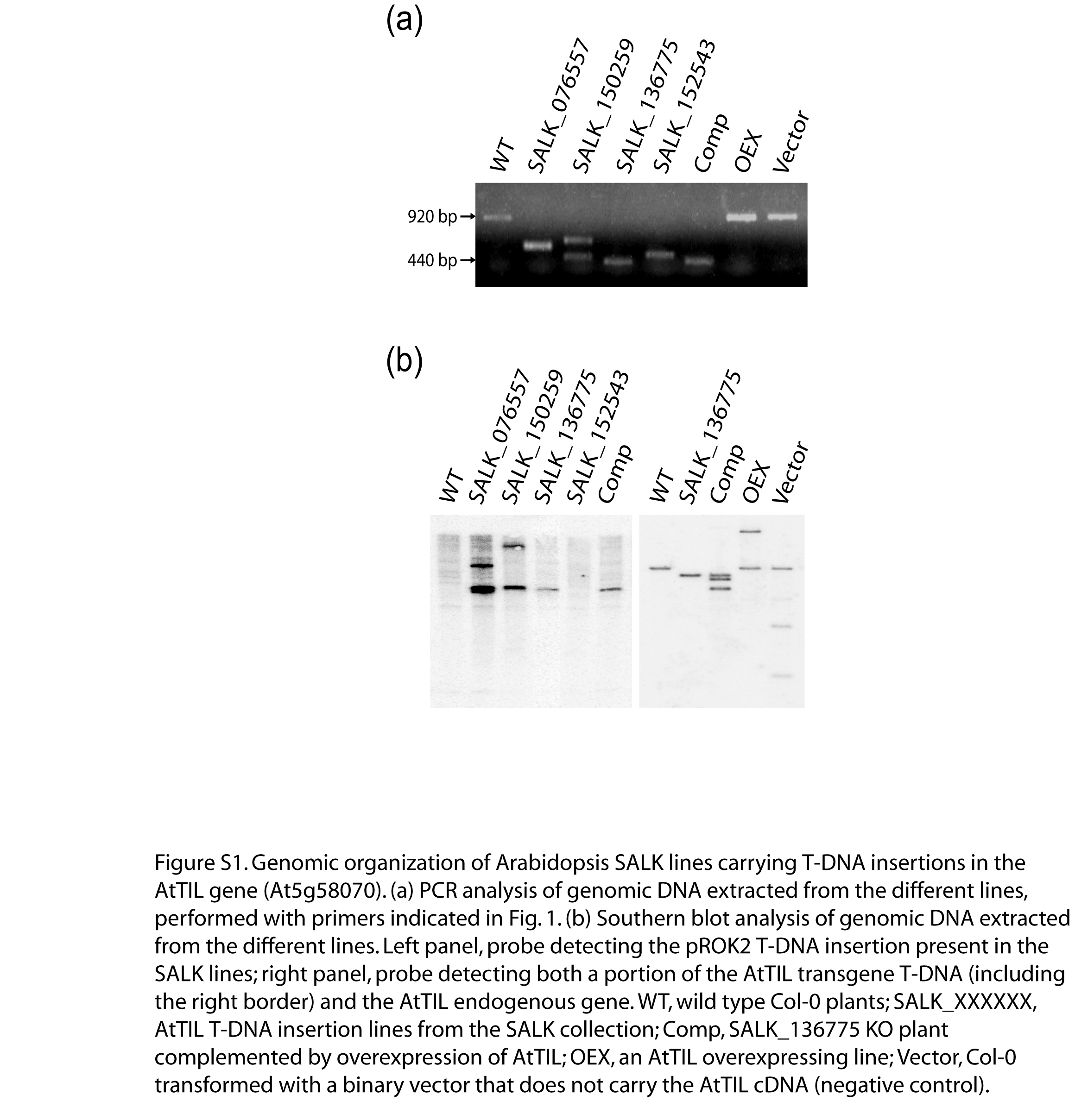

Supplement: Additional file 1 — Genomic organization of Arabidopsis SALK lines carrying T-DNA insertions in the AtTIL gene (At5g58070). (a) PCR analysis of genomic DNA extracted from the different lines, performed with primers indicated in Fig. 1. (b) Southern blot analysis of genomic DNA extracted from the different lines. Left panel, probe detecting the pROK2 T-DNA insertion present in the SALK lines; right panel, probe detecting both a portion of the AtTIL transgene T-DNA (including the right border) and the AtTIL endogenous gene. WT, wild type Col-0 plants; SALK_XXXXXX, AtTIL T-DNA insertion lines from the SALK collection; Comp, SALK_136775 KO plant complemented by overexpression of AtTIL; OEX, an AtTIL overexpressing line; Vector, Col-0 transformed with a binary vector that does not carry the AtTIL cDNA (negative control). [file 1471-2229-8-86-S1.tiff]

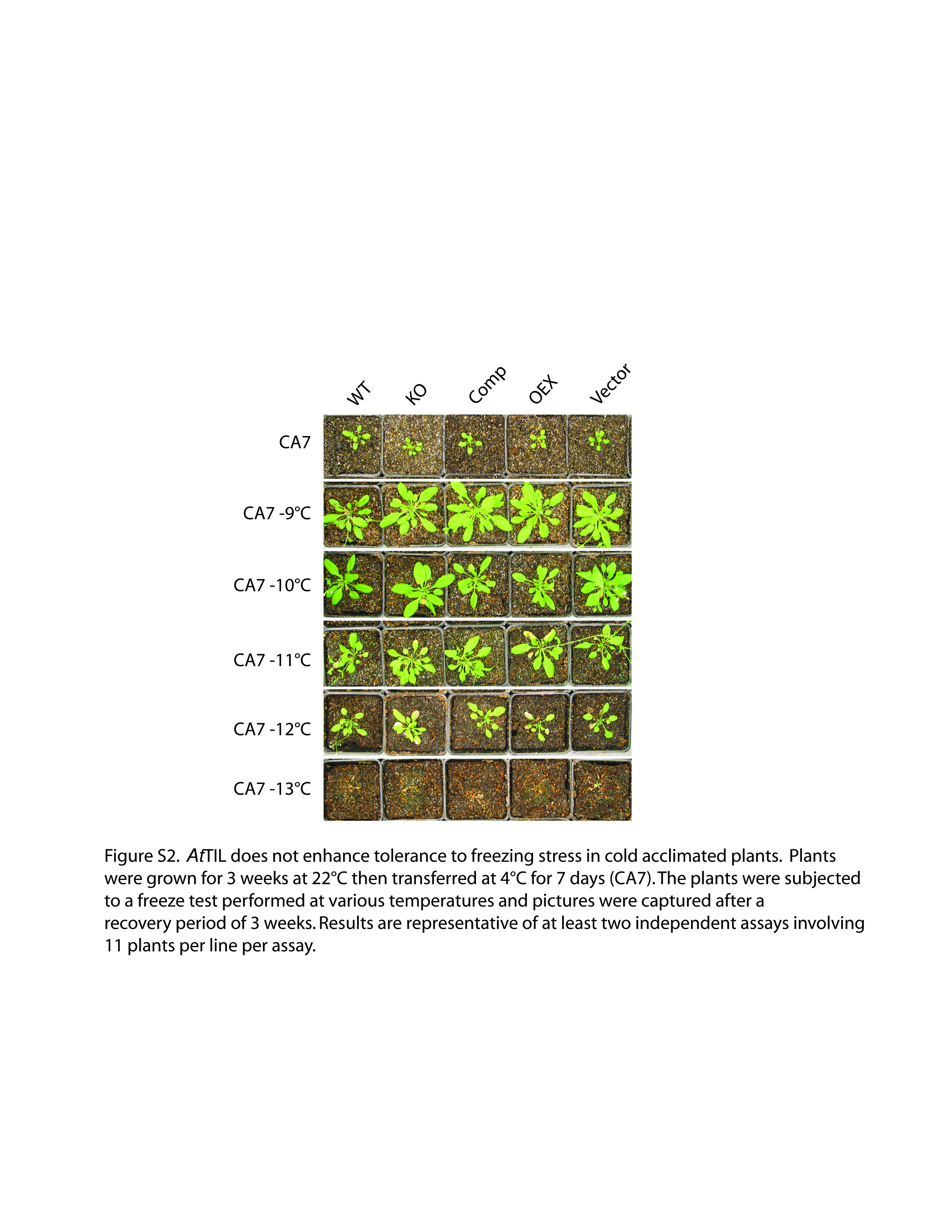

Supplement: Additional file 2 — AtTIL does not enhance tolerance to freezing stress in cold acclimated plants. Plants were grown for 3 weeks at 22°C then transferred at 4°C for 7 days (CA7). The plants were subjected to a freeze test performed at various temperatures and pictures were captured after a recovery period of 3 weeks. Results are representative of at least two independent assays involving 11 plants per line per assay. [file 1471-2229-8-86-S2.tiff]

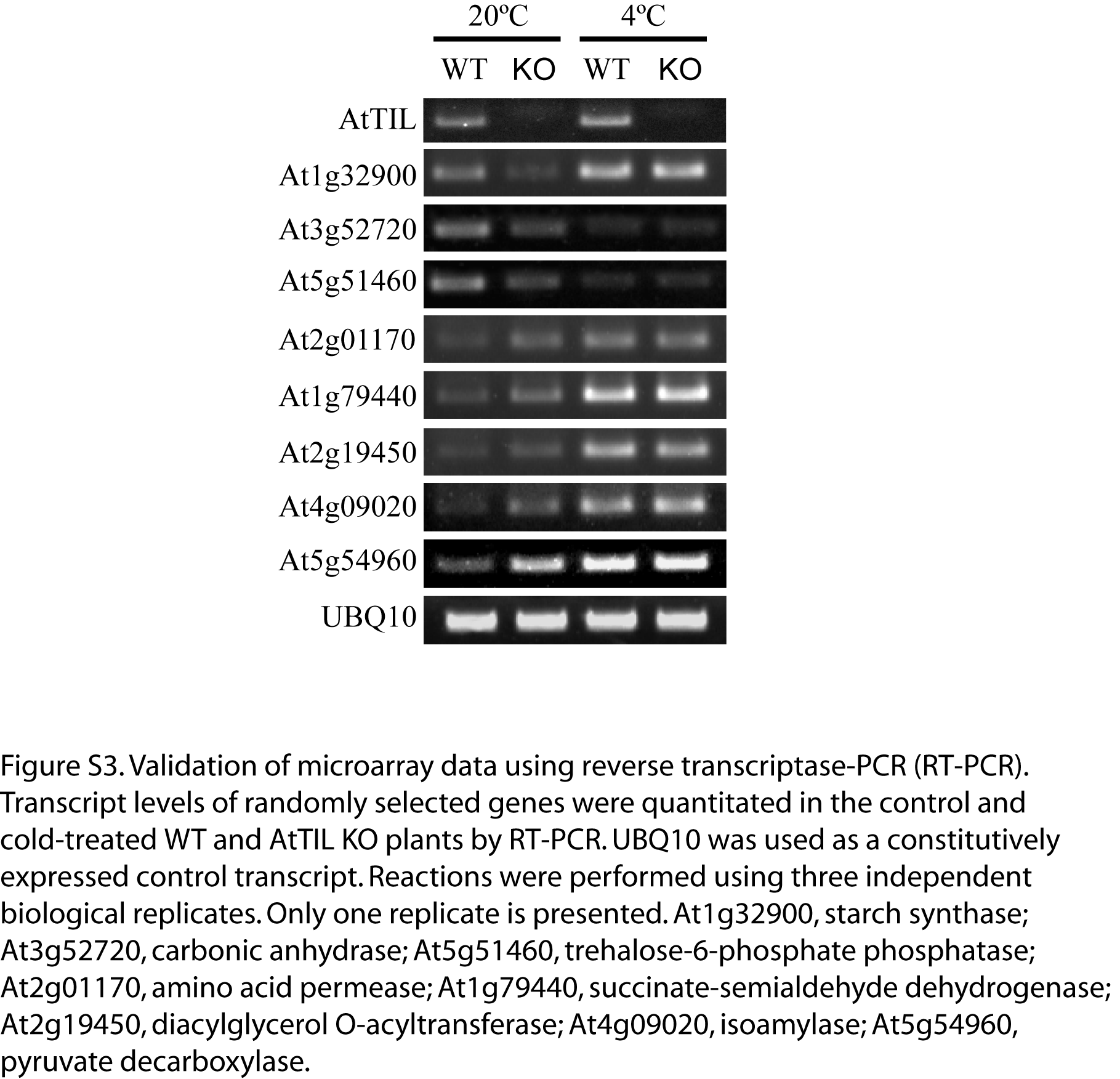

Supplement: Additional file 3 — Validation of microarray data using reverse transcriptase-PCR (RT-PCR). Transcript levels of randomly selected genes were quantitated in the control and cold-treated WT and AtTIL KO plants by RT-PCR. UBQ10 was used as a constitutively expressed control transcript. Reactions were performed using three independent biological replicates. Only one replicate is presented. At1g32900, starch synthase; At3g52720, carbonic anhydrase; At5g51460, trehalose-6-phosphate phosphatase; At2g01170, amino acid permease; At1g79440, succinate-semialdehyde dehydrogenase; At2g19450, diacylglycerol O-acyltransferase; At4g09020, isoamylase; At5g54960, pyruvate decarboxylase. [file 1471-2229-8-86-S3.tiff]
